# Supplementary material for: Clinical characteristics and risk of second primary lung cancer after cervical cancer: A population-based study
Source: PLoS One. 2020 Aug 5;15(8):e0231807. doi: 10.1371/journal.pone.0231807 (PMC7406086; doi:10.1371/journal.pone.0231807)
Supplement: Attachment — Submitted filename: Response to Reviewers.docx [file pone.0231807.s001.docx]

RE: Manuscript ID # PONE-D-20-09128

Dear Editor,

We would like to thank **PLOS ONE** for giving us the opportunity to revise our manuscript.

We thank the reviewers and editors for their careful read and thoughtful comments on previous draft. We have carefully taken their comments into consideration in preparing our revision, which has resulted in a paper that is clearer and more compelling.

**Below is our point-by-point response to the reviewer#1’s comments.**

We want to begin by thanking Reviewer #1 for positive comments . We addressed all the points raised by the reviewer, as summarized below.

1. According to the major comment ,as a negative study mentioned by the reviewer ,we have emphasized this result in the abstract（line 37 in Revised Manuscript with Track Changes） and highlighted it in the discussion section（line 267-275 in Revised Manuscript with Track Changes）.
2. As suggested by the reviewer,in order to make the discussion more concise,we have deleted discussion about age, black race and radiotherapy as risk factors for lung cancer in discussion(line 236-238 in Revised Manuscript with Track Changes)
3. According to the minor comment , as suggested by the reviewer, we have deleted the number of patients in methods section, population paragraph(line 91-92 in Revised Manuscript with Track Changes)
4. As suggested by the reviewer, we have added two references18,20(line288-290,line296-297 in Revised Manuscript with Track Changes).

**Below is our point-by-point response to the editor’s comments and additional requirements,including：**

1. we have revised the manuscript to meet the PLOS ONE's style requirements.
2. We have thoroughly copyedited our manuscript for language usage, spelling, and grammar with help of a professional scientific editing service named Mjeditor (www.mjeditor.com). A copy of my manuscript showing changes by using track changes and an editorial certificate uploaded as *supporting information* files.
3. In the Methods section, we added the accession number of the specific dataset downloaded from the SEER database for this study（line 86-87 in Revised Manuscript with Track Changes）.
4. I have authenticated a pre-existing ORCID ID in Editorial Manager.
5. We have made changes to our financial disclosure and included the updated statement in our cover letter.

Thanks for all the help.

Best wishes,

Dr Mingfang Xu

Corresponding Author

2020-06-22
